# Synthesis, Characterization, and Preclinical Evaluation of New Thiazolidin-4-Ones Substituted with p-Chlorophenoxy Acetic Acid and Clofibric Acid against Insulin Resistance and Metabolic Disorder

**DOI:** 10.1155/2014/620434

**Published:** 2014-06-03

**Authors:** Vasantharaju S. Gowdra, Jayesh Mudgal, Punit Bansal, Pawan G. Nayak, Seethappa A. Manohara Reddy, Gautham G. Shenoy, Manna Valiathan, Mallikarjuna R. Chamallamudi, Gopalan K. Nampurath

**Affiliations:** ^1^Department of Pharmaceutical Quality Assurance, Manipal College of Pharmaceutical Sciences, Manipal University, Manipal, Karnataka 576104, India; ^2^Department of Pharmacology, Manipal College of Pharmaceutical Sciences, Manipal University, Manipal, Karnataka 576104, India; ^3^Department of Pharmaceutical Chemistry, Manipal College of Pharmaceutical Sciences, Manipal University, Manipal, Karnataka 576104, India; ^4^Department of Pathology, Kasturba Medical College, Manipal University, Manipal, Karnataka 576104, India

## Abstract

We synthesized twenty thiazolidin-4-one derivatives, which were then characterized by standard chromatographic and spectroscopic methods. From the *in vitro* glucose uptake assay, two compounds behaved as insulin sensitizers, where they enhanced glucose uptake in isolated rat diaphragm. In high-carbohydrate diet-induced insulin resistant mice, these two thiazolidin-4-ones attenuated hyperglycemia, hyperinsulinemia, hypertriglyceridemia, hypercholesterolemia, and glucose intolerance. They raised the plasma leptin but did not reverse the diabetes-induced hypoadiponectinemia. Additionally, compound **3a** reduced adiposity. The test compounds were also able to reverse the disturbed liver antioxidant milieu. To conclude, these two novel thiazolidin-4-ones modulated multiple mechanisms involved in metabolic disorders, reversing insulin resistance and thus preventing the development of type-2 diabetes.

## 1. Introduction

Type-2 diabetes is a major health problem worldwide. This multifactorial disease and its complications like diabetic retinopathy, neuropathy, and nephropathy result in considerable morbidity and mortality. Diabetics are also at a greater risk to develop atherosclerosis and coronary heart disease (CHD). Metabolic syndrome characterized by dyslipidemia, hyperglycemia, insulin resistance, hypertension, and obesity also is a predisposing condition for atherosclerosis and CHD [1]. A number of drugs are routinely used to treat the various conditions of metabolic syndrome. This often leads to patient non-compliance besides raising the overall cost of treatment [2, 3]. Further, the drugs employed in treating dyslipidemia and hyperglycemia have their limitations, mainly adverse effects like weight gain and increased cardiac toxicity. Therefore, there is a need for the discovery and development of drugs with multiple actions and lower toxicity profile. This will improve the treatment outcome, reduce the number of drugs required, reduce treatment cost, and consequently, lead to greater patient compliance.

Further, recent understanding of the pathomechanism of metabolic disorder has revealed the central role of inflammation [4, 5]. Downregulation of inflammatory milieu by a hypolipidemic agent such as clofibrate through modulation of PPAR*α* provides insights into adopting the approach for synthesizing the molecules having an impact on inflammatory and metabolic machinery [6].

Thiazolidin-4-one derivatives have been extensively studied for their varied biological activities. Panetta et al. reported the anti-inflammatory effect of several 4-thiazolidinones [7]. A number of thiazolidinones have been shown to possess antioxidant and calcium overload inhibiting activities [8]. Suitably substituted thiazolidinones have been reported to reduce serum cholesterol, triglyceride, and glucose levels in our laboratory [9, 10]. Three thiazolidin-4-ones were reported to exhibit hypolipidemic activity in mice given high-fat diet and fructose [11]. Another study from our laboratory reported the antidiabetic effect of two thiazolidin-4-ones through islet cell protection in streptozotocin-induced diabetes [12]. These molecules carried a substituted benzene ring at C2 of the thiazolidine ring and nicotinoylamino moiety at the 3rd position (N) of the ring.

p-Chlorophenoxyacetic acid is structurally related to the hypolipidemic drug, clofibrate. The latter molecule is a ligand for the peroxisome proliferator activated receptor (PPAR*α*). The present work was carried out on the presumption that a clofibrate or structurally related p-chlorophenoxyacetic acid linked through an amide to the N of the thiazolidine ring would enhance the hypolipidemic and antidiabetic effect of the molecule.

In our earlier studies, a few p-chlorophenoxyacetic acid derivatives were found to possess remedial effect on cellular signalling of inflammation [13]. These molecules suppressed the inflammatory signalling via inhibition of proinflammatory mediators such as interleukin-6 (IL-6), tumor necrosis factor-*α* (TNF-*α*), and prostaglandin-E_2_ (PGE_2_). In this aspect, the proposed study was aimed to synthesize and evaluate a series of thiazolidin-4-ones from the same scaffold and to study their effects on multiple etiological factors of metabolic disorder like glucose intolerance, insulin resistance, hyperglycemia, hypertriglyceridemia, and hypercholesterolemia.

## 2. Materials and Methods

### 2.1. Chemical and Instruments

All the chemicals used in the synthesis of compounds were of analytical grade. Compounds were characterized by using various analytical instruments such as melting point apparatus, thin layer chromatography, UV/VIS and IR spectrometer, LC-MS, and RP-HPLC with the specification as reported previously [13].

### 2.2. General Synthetic Procedure for p-Chlorophenoxyacetic Acid Derivatives

**Figure 1:**
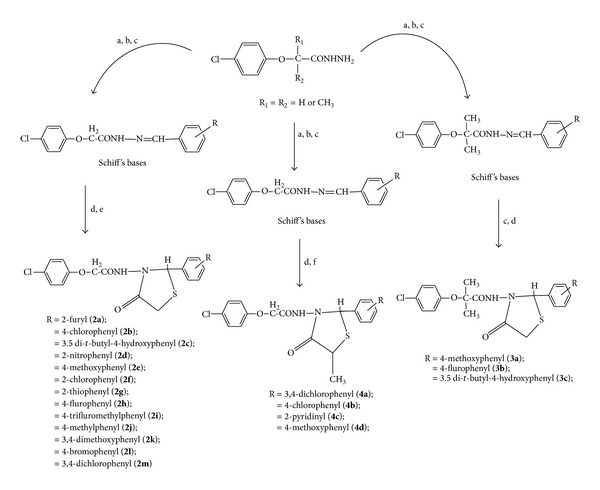
Scheme for the synthesis of thiazolidin-4-ones. Reagents and conditions: (a) aromatic aldehydes, (b) methanol, (c) glacial acetic acid, reflux for 15–45 minuts, (d) dry benzene/toluene, (e) thioglycolic acid, reflux for 24–48 hrs or microwave irradiation at power setting of 80% with 3 minutes/cycle for 16 minutes, and (f) thiolactic acid.

p-Chlorophenoxyacetic acid derivatives (Table 1) were synthesized as described previously [13], which is schematically described in Figure 1.

#### 2.2.1. 2-(4-Chlorophenoxy)-N-(2-(furan-2-yl)-4-oxothiazolidin-3-yl)acetamide (Compound 2a)

^1^HNMR (CDCl_3_) *δ*: 3.80 (s, 2H, CH_2_), 4.55 (s, 2H, CO-CH_2_-O), 5.97 (s, 1H, CH), 6.85 (m, 3H, Furan), 7.42 (m, 4H, Ar), 8.13 (br s, 1H, NH). IR (KBr) cm^−1^: 3429, 3053, 2978, 1708, 1668, 1589, 1236, 1068, 933, 827, 744, 669. UV *λ*_max⁡_ (MeoH) 279 nm. LC-MS (ESI) *m*/*z*: 353 (Calcd for C_15_H_13_ Cl N_2_O_4_S: 352.79).

#### 2.2.2. 2-(4-Chlorophenoxy)-N-(2-(4-chlorophenyl)-4- oxothiazolidin-3-yl)acetamide (Compound 2b)

^1^HNMR (CDCl_3_) *δ*: 3.70 (s, 2H, CH_2_), 4.47 (s, 2H, CO-CH_2_-O), 5.90 (s, 1H, CH), 6.90 (m, 4H, Ar), 7.36 (m, 4H, Ar), 8.0 (br s, 1H, NH). IR (KBr) cm^−1^: 3450, 3059, 2989, 1710, 1678, 1591, 1236, 1172, 1060, 962, 833, 763, 671. UV *λ*_max⁡_ (MeoH) 280 nm. LC-MS (ESI) *m*/*z*: 397 (Calcd for C_17_H_14_ Cl_2_ N_2_O_3_S: 397.28).

#### 2.2.3. 2-(4-Chlorophenoxy)-N-(2-(3,5-di-*tert*-butyl-4-hydroxyphenyl)-4-oxothiazolidin-3-yl)acetamide (Compound 2c)

^1^HNMR (CDCl_3_) *δ*: 1.49 (m, 18H, (CH_3_)_6_), 3.75 (s, 2H, CH_2_), 4.50 (s, 2H, CO-CH_2_-O), 5.40 (s, 1H, -OH), 5.90 (s, 1H, CH), 6.90 (m, 2H, Ar), 7.2 (m, 4H, Ar), 7.9 (br s, 1H, NH). IR (KBr) cm^−1^: 3620, 3327, 3064, 2056, 1726, 1685, 1595, 1246, 1155, 1058, 1008, 889, 833, 675. UV *λ*_max⁡_ (MeoHl) 278 nm. LC-MS (ESI) *m*/*z*: 491 (Calcd C_25_H_31_ Cl N_2_O_4_S: 491.04).

#### 2.2.4. 2-(4-Chlorophenoxy)-N-(2-(2-nitrophenyl)-4-oxothiazolidin-3-yl)acetamide (Compound 2d)

^1^HNMR (CDCl_3_) *δ*: 3.77 (s, 2H, CH_2_), 4.53 (s, 2H, CO-CH_2_-O), 6.4 (s, 1H, CH), 7.19 (m, 4H, Ar), 8.0 (m, 4H, Ar), 8.18 (br s, 1H, NH). IR (KBr) cm^−1^: 3427, 3072, 2914, 1722, 1680, 1597, 1521, 1386, 1242, 1170, 1063, 825, 790, 667. UV *λ*_max⁡_ (MeOH) 258 nm. LC-MS (ESI) *m*/*z*: 408 (Calcd for C_17_H_14_ Cl N_3_O_3_S: 407.83).

#### 2.2.5. 2-(4-Chlorophenoxy)-N-(2-(4-methoxyphenyl)-4-oxothiazolidin-3-yl)acetamide (Compound 2e)

^1^HNMR (CDCl_3_) *δ*: 3.85 (t, 3H, CH_3_), 3.88 (s, 2H, CH_2_), 4.65 (s, 2H, CO-CH_2_-O), 5.90 (s, 1H, CH), 7.0 (m, 4H, Ar), 7.50 (m, 4H, Ar), 8.6 (br s, 1H, NH). IR (KBr) cm^−1^: 3446, 3030, 2916, 1703, 1662, 1595, 1280, 1238, 1170, 1074, 950, 823, 763, 669. UV *λ*_max⁡_ (MeOH) 280 nm. LC-MS (ESI) *m*/*z*: 393 (Calcd for C_18_H_17_ Cl N_2_O_4_S: 392.86).

#### 2.2.6. 2-(4-Chlorophenoxy)-N-(2-(2-chlorophenyl)-4-oxothiazolidin-3-yl)acetamide (Compound 2f)

^1^HNMR (CDCl_3_) *δ*: 3.72 (s, 2H, CH_2_), 4.50 (s, 2H, CO-CH_2_-O), 6.40 (s, 1H, CH), 6.90 (m, 4H, Ar), 7.47 (m, 4H, Ar), 8.03 (br s, 1H, NH). IR (KBr) cm^−1^: 3244, 3064, 2987, 1719, 1684, 1589, 1240, 1095, 1055, 958, 825, 758, 669. UV *λ*_max⁡_ (MeOH) 279 nm. LC-MS (ESI) *m*/*z*: 397 (Calcd for C_17_H_14_ Cl_2_ N_2_O_3_S: 397.28).

#### 2.2.7. 2-(4-chlorophenoxy)-N-(4-oxo-2-(thiophen-2-yl)thiazolidin-3-yl)acetamide: (Compound 2g)

^1^HNMR (CDCl_3_) *δ*: 3.30 (d, 2H, CH_2_), 4.65 (s, 2H, COCH_2_-O), 6.90 (s, 1H, CH), 7.16 (m, 3H, Thiophen), 7.46 (m, 4H, Ar), 8.75 (br s, 1H, NH). IR (KBr) cm^−1^: 3437, 3030, 2908, 1716, 1678, 1604, 1236, 1182, 1047, 945, 823, 765, 665. UV *λ*_max⁡_ (MeOH) 327 nm. LC-MS (ESI) *m*/*z*: 369 (Calcd for C_15_H_13_ Cl N_2_O_3_S_2_: 368.86).

#### 2.2.8. 2-(4-Chlorophenoxy)-N-(2-(4-fluorophenyl)-4-oxothiazolidin-3-yl)acetamide (Compound 2h)

^1^HNMR (CDCl_3_) *δ*: 3.70 (s, 2H, CH_2_), 4.60 (s, 2H, CO-CH_2_-O), 5.90 (s, 1H, CH), 6.95 (m, 8H, Ar), 7.95 (br s, 1H, NH). IR (KBr) cm^−1^: 3209, 2922, 1710, 1678, 1599, 1230, 1163, 1070, 1008, 829, 752, 671. UV *λ*_max⁡_ (MeOH) 281 nm. LC-MS (ESI) *m*/*z*: 381 (Calcd for C_17_H_14_ Cl F N_2_O_3_S: 380.82).

#### 2.2.9. 2-(4-Chlorophenoxy)-N-(4-oxo-2-(4-(trifluoromethyl)phenyl)thiazolidin-3-yl)acetamide (Compound 2i)

^1^HNMR (CDCl_3_) *δ*: 3.89 (s, 2H, CH_2_), 4.65 (s, 2H, CO-CH_2_-O), 5.95 (s, 1H, CH), 7.86 (m, 8H, Ar), 8.00 (br s, 1H, NH). IR (KBr) cm^−1^: 3275, 3090, 2937, 1735, 1716, 1595, 1228, 1163, 1055, 960, 850, 666.UV *λ*_max⁡_ (MeOH) 283 nm. LC-MS (ESI) *m*/*z*: 431 (Calcd for C_18_H_14_ Cl F_3_ N_2_O_3_S: 430.83).

#### 2.2.10. 2-(4-Chlorophenoxy)-N-(4-oxo-2-p-tolyl thiazolidin-3-yl)acetamide (Compound 2j)

^1^HNMR (CDCl_3_) *δ*: 2.90 (s, 3H, CH_3_), 3.70 (s, 2H, CH_2_), 4.42 (s, 2H, CO-CH_2_-O), 5.90 (s, 1H, CH), 6.95 (m, 7H, Ar), 8.05 (br s, 1H, NH). IR (KBr) cm^−1^: 3217, 2989, 1722, 1676, 1589, 1292, 1238, 1220, 1170, 1060, 962, 821, 740, 667. UV *λ*_max⁡_ (MeOH) 279 nm. LC-MS (ESI) *m*/*z*: 377 (Calcd for C_18_H_17_ Cl N_2_O_3_S: 376.86).

#### 2.2.11. 2-(4-Chlorophenoxy)-N-(2-(3,4-dimethoxyphenyl)-4-oxothiazolidin-3-yl)acetamide (Compound 2k)

^1^HNMR (CDCl_3_) *δ*: 3.85 (s, 2H, CH_2_), 3.88 t, 6H, [(OCH_3_)_2_], 4.20 (s, 2H, CO-CH_2_-O), 4.59 (s, 2H, CH_2_), 5.89 (s, 1H, CH), 7.20 (m, 7H, Ar), 8.00 (br s, 1H, NH). IR (KBr) cm^−1^: 3448, 3026, 2974, 1703, 1662, 1593, 1236, 1072, 950, 821, 796, 669. UV *λ*_max⁡_ (MeOH) 279 nm. LC-MS (ESI) *m*/*z*: 423 (Calcd for C_19_H_19_Cl N_2_O_5_S: 422.88).

#### 2.2.12. N-(2-(4-Bromophenyl)-4-oxothiazolidin-3-yl)-2-(4-chlorophenoxy)acetamide (Compound 2l)

^1^HNMR (CDCl_3_) *δ*: 3.05 (s, 2H, CH_2_), 3.78 (s, 2H, CO-CH_2_-O), 5.64 (s, 1H, CH), 6.72 (m, 4H, Ar), 7.15 (m, 4H, Ar), 7.84 (br s, 1H, NH). IR (KBr) cm^−1^: 3448, 3051, 2982, 1714, 1674, 1587, 1230, 1176, 1058, 964, 829 & 673. UV *λ*_max⁡_ (MeOH) 278 nm. LC-MS (ESI) *m*/*z*: 442 (Calcd for C_17_H_14_Br Cl N_2_O_3_S: 441.73).

#### 2.2.13. 2-(4-Chlorophenoxy)-N-(2-(3,4-dichlorophenyl)-4-oxothiazolidin-3-yl)acetamide (Compound 2m)

^1^HNMR (CDCl_3_) *δ*: 3.29 (s, 2H, -CH_2_), 3.88 (s, 2H, -CH_2_-CO), 5.64 (s, 1H, CH), 6.82 (m, 3H, Ar), 7.33 (m, 4H, Ar), 8.52 (br s, 1H, NH). IR (KBr) cm^−1^: 3228, 3022, 2914, 1730, 1687, 1585, 1481, 1369, 1219, 1130, 1060, 960, 823, 758, 667. UV *λ*_max⁡_ (MeOH) 279 nm. LC-MS (ESI) *m*/*z*: 432 (Calcd for C_17_H_13_ Cl_3_ N_2_O_3_S: 431.72).

#### 2.2.14. 2-(4-Chlorophenoxy)-N-(2-(3,4-dichlorophenyl)-5-methyl-4-oxothiazolidin-3-yl)acetamide (Compound 4a)

^1^HNMR (CDCl_3_) *δ*: 1.53 (s, 3H, -CH_3_), 3.67 (1H, -CH-CH_3_), 3.90 (s, 2H, -CH_2_-), 5.68 (s, 1H, -CH), 6.95 (m, 3H, Ar), 7.25 (m, 4H, Ar′), 8.36 (br s, 1H, -NH). IR (KBr) cm^−1^: 3277, 3050, 2980, 1726, 1695, 1585, 1490, 1378, 1217, 1126, 1060, 966, 821, 738, 638. UV *λ*_max⁡_ (MeOH) 278 nm. LC-MS (ESI) *m*/*z*: 448 (Calcd for C_18_H_15_ Cl_3_ N_2_O_3_S: 445.75).

#### 2.2.15. 2-(4-Chlorophenoxy)-N-(2-(4-chlorophenyl)-5-methyl-4-oxothiazolidin-3-yl)acetamide (Compound 4b)

^1^HNMR (CDCl_3_) *δ*: 1.55 (s, 3H, -CH_3_), 3.40 (1H, -CH-CH_3_), 3.92 (s, 2H, -CH_2_-), 5.69 (s, 1H, -CH), 6.77 (m, 4H, Ar), 7.03 (m, 4H, Ar′), 7.66 (br s, 1H, -NH). IR (KBr) cm^−1^: 3279, 3057, 2980, 1726, 1693, 1585, 1383, 1205, 1168, 1070, 962, 773, 636. UV *λ*_max⁡_ (MeOH) 279 nm. LC-MS (ESI) *m*/*z*: 411 (Calcd for C_18_H_16_ Cl_2_ N_2_O_3_S: 411.30).

#### 2.2.16. 2-(4-Chlorophenoxy)-N-(5-methyl-4-oxo-2-(pyridin-2-yl)thiazolidin-3-yl)acetamide (Compound 4c)

^1^HNMR (CDCl_3_) *δ*: 1.45 (s, 3H, -CH_3_), 3.55 (1H, -CH-CH_3_), 3.89 (s, 2H,-CH_2_), 6.10 (s, 1H, -CH), 6.52 (m, 4H, Pyridine), 6.90 (m, 4H, Ar), 8.45 (br s, 1H, -NH). IR (KBr) cm^−1^: 3159, 2966, 1732, 1691, 1587, 1496, 1342, 1238, 1180, 1060, 997, 829, 754, 661. UV *λ*_max⁡_ (MeOH) 263 nm. LC-MS (ESI) *m*/*z*: 378 (Calcd for C_17_H_16_ Cl N_3_O_3_S: 377.85).

#### 2.2.17. 2-(4-Chlorophenoxy)-N-(2-(4-methoxyphenyl)-5-methyl-4-oxothiazolidin-3-yl)acetamide (Compound 4d)

^1^HNMR (CDCl_3_) *δ*: 3.24 (s, 3H, -CH_3_), 3.97 (s, 3H, -CH-CH_3_), 5.94 (s, 1H, -CH), 6.66 (m, 4H, Ar), 7.15 (m, 4H, Ar′), 8.15 (br s, 1H, -NH). IR (KBr) cm^−1^: 3281, 2935, 1735, 1683, 1587, 1383, 1244, 1165, 1064, 964, 821, 744, 636. UV *λ*_max⁡_ (MeOH) 280 nm. LC-MS (ESI) *m*/*z*: 407 (Calcd for C_19_H_19_ Cl N_2_O_4_S: 406.88).

### 2.3. General Synthetic Procedure for 2-(4-Chlorophenoxy)-2-Methylpropanoic Acid Derivatives [14]

Ethyl-2-(4-chlorophenoxy)-2-methylpropanoate was synthesized by esterifying 2-(4-chlorophenoxy)-2-methylpropanoic acid (clofibric acid, 0.06 mol) with ethanol. The ester was converted into hydrazide by reacting ethyl 2-(4-chlorophenoxy)-2-methylpropanoate (0.1 mol) with hydrazine hydrate (0.1 mol). Schiff's bases were prepared by reacting 2-(4-chlorophenoxy)-2-methylpropanehydrazide (0.1 mol) with Ar aldehydes (0.1 mol) like anisaldehyde, p-fluorobenzaldehyde, and 3,5-di-*tert*-butyl-4-hydroxybenzaldehyde in presence of 4-5 drops of glacial acetic acid.

Schiff's base (0.01 mol) and thioglycolic acid (0.02 mol) were refluxed in dry benzene. The water formed during cyclization was removed azeotropically. The completion of the reaction was checked chromatographically. The period of reflux varied from compound to compound. After the completion of the reaction, the solvent was distilled off under reduced pressure. The reaction mixture was poured into water containing sodium bicarbonate solution. The solid separated was filtered, dried, and recrystallized from methanol, as described schematically in Figure 1.

#### 2.3.1. 2-(4-Chlorophenoxy)-N-(2-(4-methoxyphenyl)-4-oxothiazolidin-3-yl)-2-methylpropanamide (Compound 3a)

^1^HNMR (CDCl_3_); *δ* = 1.37 (t, 3H, -CH_3_), 1.47 (t, 3H, - CH_3_), 3.70 (t, 3H, OCH_3_), 3.84 (s, 2H, CH_2_), 5.90 (s, 1H, CH), 7.35 (m, 8H, Ar), 8.17 (br s, 1H, -NH) ppm; IR (KBr); 3281 (-NH), 3080 (Ar, C-H, Str), 2892, 1710 & 1680 (-C=O of -CH_2_-C=O & -CONH), 1612 (C=C, Str), 1251 (C-O, Str), 1143, 1095 (C-Cl), 848, 817, 709 & 663 (C-S-C) cm^−1^, respectively; MS (ESI): *m*/*z* = 421.

#### 2.3.2. 2-(4-Chlorophenoxy)-N-(2-(4-fluorophenyl)-4-oxothiazolidin-3-yl)-2-methylpropanamide (Compound 3b)

^1^HNMR (CDCl_3_); *δ* = 1.37 (t, 3H, -CH_3_), 1.47 (t, 3H, -CH_3_), 3.85 (s, 2H, CH_2_), 5.90 (s, 1H, CH), 7.43 (m, 8H, Ar), 8.10 (br s, 1H, -NH) ppm; IR (KBr); 3250 (-NH), 3043 (Ar, C-H, Str), 2985, 1726 & 1674 (-C=O of -CH_2_-C=O & -CONH), 1599 (C=C, Str), 1228 (C-O, Str), 1157 (C-F), 1085 (C-Cl), 937, 850 & 648 (C-S-C) cm^−1^, respectively; MS (ESI): *m*/*z* = 409.

#### 2.3.3. 2-(4-Chlorophenoxy)-N-(2-(3,5-di-*tert*-butyl-4-hydroxyphenyl)-4-oxothiazolidin-3-yl)-2-methylpropanamide (Compound 3c)

^1^HNMR (CDCl_3_); *δ* = 1.28 (s, 6H, (CH_3_)_2_), 1.42 (s, 18H, (CH_3_)_6_), 3.44 (s, 2H, -CH_2_-CO-), 4.54 (s, 1H, -OH), 5.17 (s, 2H, Ar), 6.23 (s, 1H, CH), 7.57 (m, 4H, Ar) ppm; IR (KBr); 3636 (-OH), 3543 (-NH), 3001 (Ar, C-H, Str), 2955, 1740 & 1697 (-C=O of -CH_2_-C=O & -CONH), 1600 (C=C, Str), 1236 (C-O, Str), 1209, 1024 (C-Cl), 887, 773 & 626 (C-S-C) cm^−1^, respectively; MS (ESI): *m*/*z* = 518.

### 2.4. Experimental Subjects

Male Swiss albino mice (22–25 g) and Wistar rats (220–250 g) (inbred in Central Animal Research Facility, Manipal University, Manipal, Karnataka, India) were used. At a temperature of 25 ± 0.5°C, animals were housed in plastic cages with 12 h light/dark cycle and humidity 50 ± 5% RH. Animals were given standard food pellet and water* ad libitum*. The experimental protocols were approved by the Institutional Animal Ethics Committee (no. IAEC/KMC/25/2009-2010) and the experiments were carried out in accordance with the guidelines provided by the Committee for the Purpose of Control and Supervision of Experiments on Animals (CPCSEA), Government of India.

### 2.5. *In Vitro* Glucose Uptake Assay [15]

Overnight fasted male Wistar rats (220–250 g) were sacrificed and diaphragms were isolated avoiding trauma and divided into two hemidiaphragms. After isolation, blood clots were removed by rinsing the hemidiaphragms in cold Tyrode's solution (without glucose) and transferred to the mammalian organ bath containing Tyrode's solution with 0.5% w/v glucose with/without the test/standard drug (1 mM) followed by incubation for 45 min at 37 ± 1°C in presence of aeration. After the incubation, the glucose content of the incubated organ bath was measured spectrophotometrically using colorimetric kits (Aspen Laboratories Pvt. Ltd., New Delhi, India). The difference between the initial and final glucose amount was considered as amount of glucose uptake (mg/g of tissue weight/45 min).

The following groups were made for screening the compounds; Group 1: 5 mL of Tyrode's solution with 0.5% w/v glucose (glucose control, in absence of insulin); Group 2: 5 mL of Tyrode's solution with 0.5% w/v glucose and regular insulin (Novo Nordisk India Pvt. Ltd., Bangalore, India, 40 IU/mL) 25 *μ*L containing 0.5 units of insulin (glucose control, in presence of insulin); Groups 3 to 23: 5 mL of Tyrode's solution with 0.5% w/v glucose and 1 mM test compound (total numbers = 20); Group 24: 5 mL of Tyrode's solution with 0.5% w/v glucose + 1 mM pioglitazone (standard); Group 25 to 45: 5 mL of Tyrode's solution with 0.5% w/v glucose + regular insulin 25 *μ*L containing 0.5 units of insulin + 1 mM test drug (total numbers = 20); Group 46: 5 mL of Tyrode's solution with 0.5% w/v glucose + regular insulin 25 *μ*L containing 0.5 units of insulin + 1 mM pioglitazone (standard).

### 2.6. Acute Oral Toxicity Study

An acute toxicity study was performed on Swiss albino mice according to OECD 420 guidelines according to the methods described earlier [13]. All the newly synthesized test compounds were administered at the dose of 2000 mg/kg, p.o. to the animals and observed for any sign of toxicity as described earlier [13].

### 2.7. High Carbohydrate Diet- (HCD-) Induced Metabolic Disorder in Mice [16]

Four-week-old male Swiss albino mice were placed on the feeding of high sucrose diet (HCD). The detailed composition of diet where 55% energy source (kilocalories) was from sucrose is given in Table 2.

After 24 weeks of HCD feeding, mice displayed hyperglycemia. Animals with similar degrees of hyperglycemia were randomly divided into six groups (*n* = 6). The normal pellet diet (NPD) fed mice were used as nondiabetic controls. The diabetic control (HCD) and the normal control (NPD) groups received the vehicle (0.25% CMC, 10 mL/kg), while the treatment groups were given pioglitazone (5 mg/kg, p.o.) and compound** 2e** and compound** 3a** (100 mg/kg, p.o.) respectively, suspensions in 0.25% w/v CMC (10 mL/kg). All the treatments were given for 30 days. Animals' body weight and cumulative food intake were recorded periodically. Food efficiency ratio (FER) was calculated according to the reported method [17] using the following formula: FER = change in body weight (day 0–day 30)/cumulative food consumed in 30 days per animal. Final body weight on day 30 and FER were reported. Plasma glucose, triglycerides, and total cholesterol were monitored on day 14 and day 30 after the treatment using colorimetric kits (Aspen Laboratories Pvt. Ltd., New Delhi, India). Plasma insulin (Linco Research Inc., St. Charles, MO, USA), leptin (BioVendor LLC, Candler, NC, USA), and adiponectin (Adipogen Corporation, San Diego, CA, USA) were estimated on day 30 using ELISA kits according to manufacturer's instructions. OGTT was performed on day 32 as described below. On day 32, animals were sacrificed and liver, pancreas, and white adipose tissues (from epididymal fat, WAT) were isolated for histopathological investigation.

#### 2.7.1. Oral Glucose Tolerance Test (OGTT) in Diabetic Mice [18]

OGTT was performed according to the method described previously [18]. In brief, animals were fasted overnight and distilled water or glucose load of 2 g/kg, p.o. was administered and blood samples were collected by retroorbital plexus puncture at 0, 30, 60, and 120 min after glucose challenge. Plasma glucose was measured spectrophotometrically using commercially available colorimetric kits (Aspen Laboratories Pvt. Ltd., New Delhi, India). The percentage reduction in glucose excursion (AUC_0−120 min⁡_) produced by test compounds was calculated from the area under the curve (AUC_0−120 min⁡_). The results are expressed in time-dependent plasma glucose (mg/dL) levels, plasma glucose (AUC_0−120 min⁡_), and % reduction in glucose excursion (AUC_0−120 min⁡_).

#### 2.7.2. Endogenous Liver Antioxidant Enzymes Estimation

Animals were sacrificed by cervical dislocation on the 32nd day after treatment. Transcardial and whole liver perfusion were performed using ice-cold saline [19]. Liver was isolated, and 10% w/v homogenate was prepared using Teflon-glass homogenizer (RQ-127A/D, REMI Group, Mumbai, India) with ice-cold saline-EDTA. The homogenate was centrifuged at 10,000 rpm for 10 min; supernatant was collected and centrifuged again at 20,000 rpm for 1 h at 4°C. The supernatant obtained was used for the estimation of glutathione (GSH), glutathione-S-transferase (GST), catalase, superoxide dismutase (SOD), and malondialdehyde (MDA). All the data presented as mean ± S.E.M. (*n* = 6).

*(i) Glutathione (GSH) Assay [20].* From the liver homogenate, proteins were precipitated by 10% tri-carboxylic acid (TCA) and then centrifuged to collect the supernatant. One mL supernatant was mixed with 6 mL 0.2 M pH 8.0 and 1 mL 0.6 mM 5,5′-dithiobis-(2-nitrobenzoic acid) (DTNB) and incubated for 10 min at room temperature. The absorbance was recorded against the blank at 412 nm in a UV-visible spectrophotometer (model UV-1650PC, Shimadzu Co., Kyoto, Japan) and the GSH concentration was calculated from the standard curve.

*(ii) Superoxide Dismutase Assay [21].* The entire 1 mL supernatant was added to 0.1 M carbonate buffer (pH 10.2) and the increase in absorbance after addition of epinephrine was measured at 480 nm using a UV-visible spectrophotometer (model UV-1650PC, Shimadzu Co., Kyoto, Japan). The enzyme activity was expressed as U/mg protein.

*(iii) Catalase Assay [22].* The catalase activity was determined spectrophotometrically according to the protocol of Claiborne [22]. The reaction was started by adding 0.05 mL supernatant to the reaction mixture (1.95 mL 10 mM H_2_O_2_ in 60 mM phosphate buffer, pH 7.0). Absorbance was recorded for 3 min at 240 nm. Phosphate buffer (60 mM, pH 7.0) was kept as a reference. To determine the specific activity of catalase, extinction coefficient of 0.04 mM^−1 ^cm^−1^ was used.

*(iv) Glutathione-S-Transferase (GST) Assay [23].* 0.1 mL 1-chloro-2,4-dinitrobenzene (CDNB) was added to 0.6 mL supernatant of liver homogenate and 2.2 mL phosphate buffer pH 6.5, incubated at 37°C for 5 min, and added 0.1 mL 30 mM GSH. Absorbance was recorded at 340 nm at intervals of 1,2,3,4,5 min. Blank was carried out in the same manner without homogenate.

*(v) Malondialdehyde Assay [24].* One mL of liver homogenate was combined with 2 mL of reaction mixture [15% w/v trichloroacetic acid (TCA) and 0.375% w/v thiobarbituric acid (TBA) in 0.25 N hydrochloric acid (HCl)] and mixed thoroughly. The solution was heated for 20 min on boiling water bath. Samples were cooled and centrifuged at 1000 rpm for 10 min to remove the flocculent precipitate. Supernatant was collected and the absorbance of read at 532 nm against a blank, which contained all the reagents except the liver homogenate. The extinction coefficient of 1.56 × 105 M^-1 ^cm^−1^ was used to calculate the malondialdehyde concentration.

#### 2.7.3. Histopathological Investigation

For histological examinations, liver, pancreas and white adipose tissue (WAT) from three animals per group were isolated on day 32 after treatment of compounds. The tissue samples were fixed in formalin solution (10%) for one week at room temperature, dehydrated by graded ethanol, cleared using graded xylene, and embedded in paraffin wax. 5 *μ*m thick sections were cut using rotary microtome (RM2245, Leica Microsystems GmbH, Wetzlar, Germany), fixed on glass slides, stained with eosin and hematoxylin, and observed using a microscope (Model BX41, Olympus Corporation, Tokyo, Japan). For Langerhans cells, the average areas of six islets per specimen were measured using ImageJ software (version 1.48, National Institute of Health, MD, USA). Area was expressed as *μ*m^2^. The quantification of adipocytes were performed as reported [25], using adipocyte quantification tool, where area was measured in *μ*m^2^.

### 2.8. Statistical Analysis

Statistical analysis was performed by comparing the responses of the treatment groups to respective saline control and vehicle treated groups for all experiments and the significance was determined by one-way ANOVA followed by post hoc Dunnett's test. Values were expressed as mean ± S.E.M. *P* < 0.05 was considered significant.

## 3. Results

### 3.1. *In Vitro* Biological Study

#### 3.1.1. Glucose Uptake Assay

Of the twenty thiazolidin-4-ones tested, compounds** 2e** and** 3a **stimulated glucose uptake (*P* < 0.05) when compared with control. Uptake was stimulated both in the absence and presence of external insulin. The standard drug, pioglitazone, also increased the glucose uptake (Table 3).

### 3.2. *In Vivo* Biological Studies

#### 3.2.1. Acute Oral Toxicity Study

Acute toxicity studies were carried out on Swiss albino mice as per the OECD guidelines for test compounds** 2e** and** 3a**. Both were found to be safe up to 2000 mg/kg which was the maximum dose tested.

#### 3.2.2. High Carbohydrate Diet- (HCD-) Induced Metabolic Disorder in Mice

Animals put on HCD for 6 months had significant hyperglycemia, hypertriglyceridemia, and hypercholesterolemia. They were randomized to different treatment groups based on plasma glucose level and then treatment was given for 30 days. Firstly, treatment did not have any significant effect on body weight of animals (Table 4). Secondly, energy expenditure was assessed indirectly using FER. None of the treated groups except compound** 3a**-treated group had any significant effect on FER. The calculated FER was found to be significantly higher in animal treated with compound** 3a** compared to normal control and HCD group. High FER value indicates the increased energy expenditure in animals treated with compound** 3a**.

**Figure 2:**
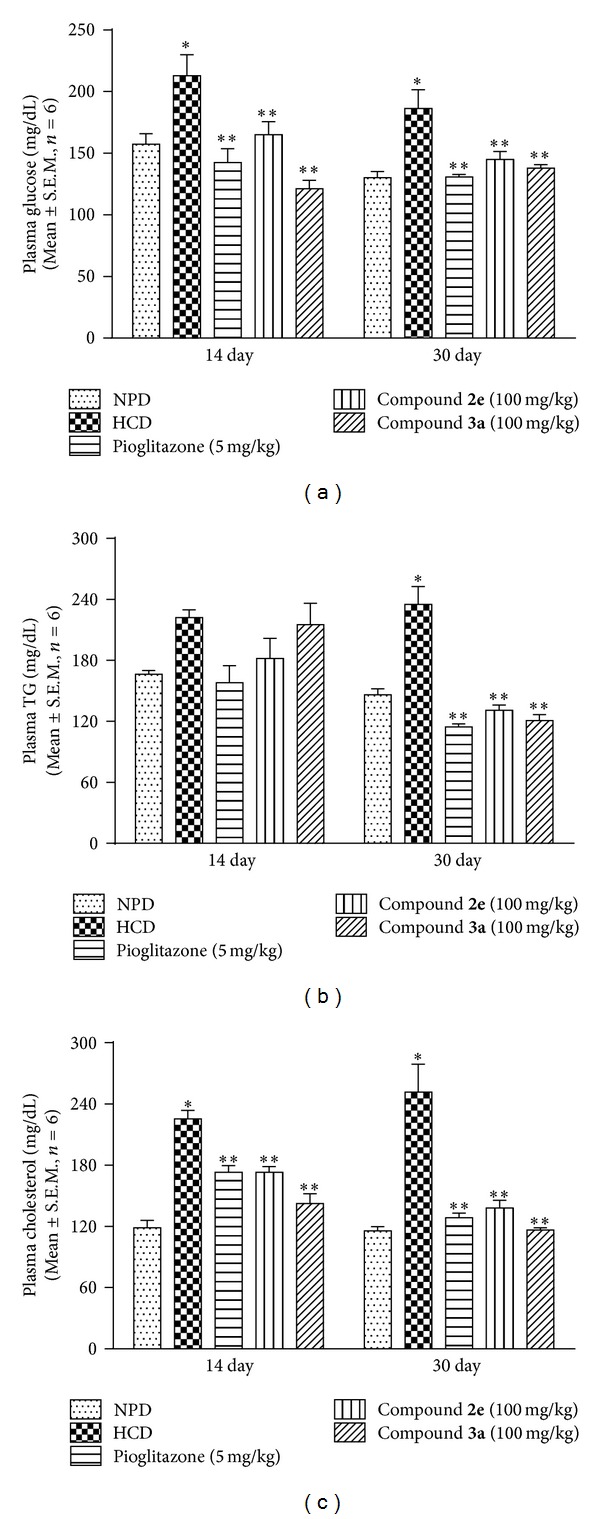
Effect of thiazolidin-4-ones on plasma: (a) glucose; (b) triglyceride (TG); (c) cholesterol in HCD fed mice. Data presented as mean ± S.E.M. (*n* = 6).  *represents *P* < 0.05 as compared to NPD group and  **represents *P* < 0.05 as compared to HCD group.

*(i) Effect of Thiazolidin-4-Ones on Plasma Glucose, Triglyceride, and Cholesterol Level.* Biochemical estimation of metabolic markers such as plasma glucose (PG), triglycerides (TG), and total cholesterol (TC) was performed on day 14 and day 30 after the drug treatment and it was observed that HCD control group consistently had significant increase in PG and TC levels on days 14 and 30 compared to respective day's normal control group. However, TG levels were significantly increased only on day 30 compared to respective day's normal control group. Pioglitazone and test compounds (**2e** and** 3a)** significantly reversed hyperglycemia and elevated plasma cholesterol compared to HCD control group on both day 14 and day 30 of drug treatment (Figures 2(a) and 2(c)) while elevated TG levels were reduced on day 30 by the test compounds and pioglitazone (Figure 2(b)).

**Figure 3:**
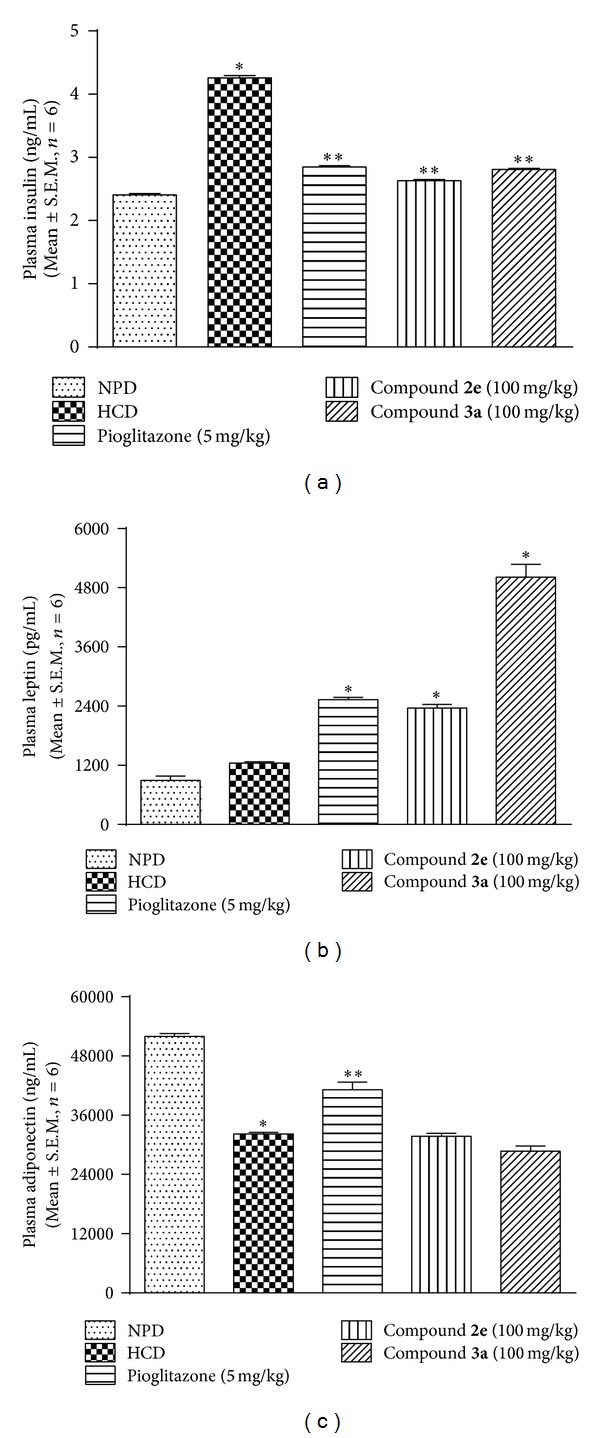
Effect of thiazolidin-4-ones on plasma: (a) insulin; (b) leptin; (c) adiponectin in HCD fed mice. Data presented as mean ± S.E.M. (*n* = 6).  *represents *P* < 0.05 as compared to NPD group and  **represents *P* < 0.05 as compared to HCD group.

*(ii) Effect of Thiazolidin-4-Ones on Plasma Insulin, Leptin, and Adiponectin Level.* Development of insulin resistance in mouse was confirmed by estimating the plasma insulin levels on day 30 after drug treatment. HCD control group, in contrast to NPD group, showed two times increase in plasma insulin levels (*P* < 0.05). Hyperinsulinemia associated with hyperglycaemia and hypertriglyceridemia is considered to be the sign of development of insulin resistance. Thus, high sucrose feeding for 6 months led to the development of insulin resistance in mice. Treatment with pioglitazone and test drugs attenuated the insulin resistance (Figure 3(a)).

HCD feeding to animals did not elicit any significant effect on plasma leptin levels as there was no significant difference between HCD control and NPD group. However, treatment with pioglitazone and test compounds significantly (*P* < 0.05) raised the circulating leptin level in mice compared with HCD control group (Figure 3(b)). Compound** 3a** caused a fourfold elevation in leptin levels compared with HCD group. HCD feeding to animals resulted in significant (*P* < 0.05) hypoadiponectinemia compared with NPD. Treatment with pioglitazone significantly (*P* < 0.05) corrected the hypoadiponectinemia. However, both test compounds failed to correct hypoadiponectinemia (Figure 3(c)).

**Figure 4:**
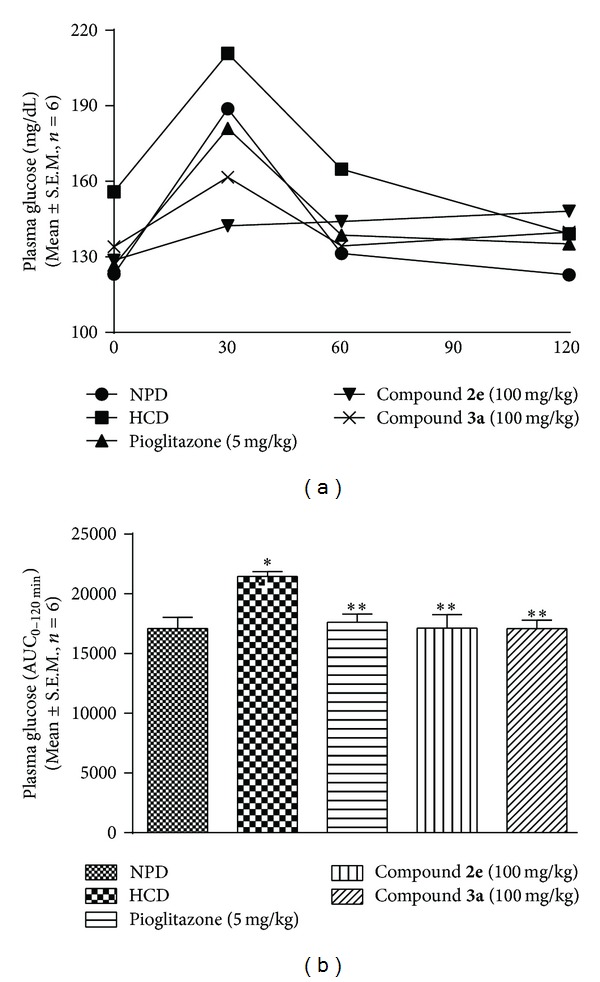
Effect of thiazolidin-4-ones on (a) plasma glucose (mg/dL) and (b) area under the curve (AUC_0−120 min⁡_) against oral glucose tolerance test (OGTT) in HCD fed mice. Data presented as mean ± S.E.M. (*n* = 6).  *represents *P* < 0.05 as compared to NPD group and  **represents *P* < 0.05 as compared to HCD group.

*(iii) Effect of Thiazolidin-4-Ones on Oral Glucose Tolerance Test.* In oral glucose tolerance test, HCD group animal showed significant (*P* < 0.05) glucose intolerance (Figure 4). Pioglitazone and test compounds, compound** 2e** and compound** 3a**, corrected the glucose intolerance, shown as significant (*P* < 0.05) percent reductions in glucose excursion (AUC_  0−120min⁡_) by 13.00 ± 3.3, 15.46 ± 5.54, and 15.60 ± 3.49, respectively, compared with HCD group (Figures 4(a) and 4(b)).

*(iv) Effect of Thiazolidin-4-Ones on Liver Enzymes.* Oxidative stress is the hallmark of metabolic disorder, where disturbed homeostasis between oxidative and antioxidative mechanism occurs. HCD feeding to mice resulted in an oxidative stress observed as reduction in liver antioxidant enzymes such as glutathione (GSH), catalase (CAT), superoxide dismutase (SOD), and glutathione-S-transferase (GST). Treatment with pioglitazone and test compounds ameliorated the oxidative stress. They also reversed the elevation in liver malondialdehyde (MDA) levels in HCD-fed mice (Table 5).

**Figure 5:**
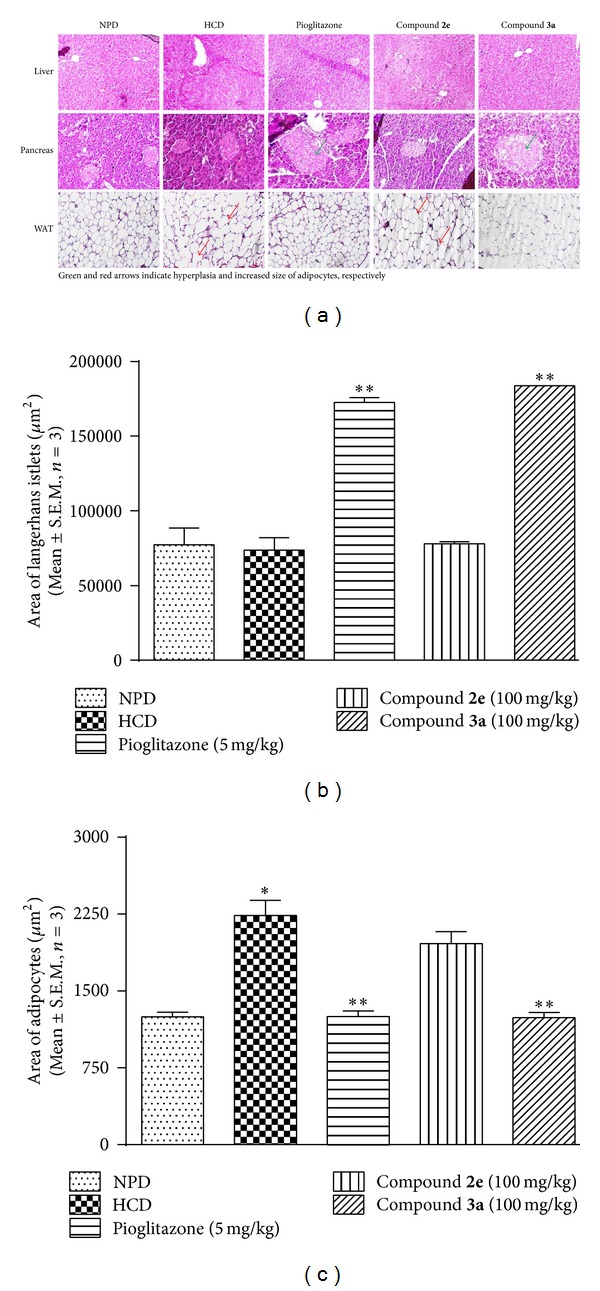
(a) Histological sections of mouse liver, pancreas, and white adipose tissue (WAT) in HCD model (10x). Green and red arrows indicate hyperplasia of islets of Langerhans and increase in the size of adipocytes, respectively. Effect of thiazolidin-4-ones on (b) area of Langerhans islets (*μ*m^2^) and (c) area of adipocytes (*μ*m^2^).  *represents *P* < 0.05 as compared to NPD group and  **represents *P* < 0.05 as compared to HCD group.

*(v) Histopathological Examination.* Histology of liver showed normal lobular architecture with normal hepatocytes in all groups (Figure 5(a)). Pancreas showed mild to moderate hyperplasia of islets of Langerhans (Figure 5(a)) in pioglitazone and compound** 3a** groups. Pioglitazone and** 3a** treatment significantly raised the area of Langerhans islets compared with HCD group (Figure 5(b)). Exocrine portion of the pancreas and vascularity appeared normal in all the groups. Histopathological investigation of white adipose tissue (WAT) from epidydimal showed an increase in the size of adipocytes (Figure 5(c)) in HCD group which was reversed by pioglitazone and compound** 3a** treatment. However, compound** 2e** did not show any significant effect on increased adipocyte size.

## 4. Discussion and Conclusion

The present work was planned as a sequel to earlier studies in our laboratory using thiazolidin-4-ones as antidiabetic, hypolipidemic, and antiinflammatory molecules. The substitutions in the thiazolidine ring were made at C2 and N3. Attached to the latter position was a nicotinamide moiety and the substitution at C2 was either p-methoxyphenyl or 2, 5-di-*tert*-butyl-4-hydroxyphenyl group. The compounds showed significant antidiabetic and hypolipidemic activities [9–11, 26].

In the present study, the substitution at N3 was changed to p-chloro-phenoxyacetylamino, a group that is similar to clofibrate with a methylene bridge instead of* gem*-dimethyl substitution.

Four compounds out of 20 (i.e., compounds** 4a**,** 4b**,** 4c**, and** 4d**) had a methyl group attached at the C5 of thiazolidine ring, with cyclisation being made with thiolactic acid instead of thioglycolic acid. In three other compounds (i.e., compounds** 3a**,** 3b**, and** 3c**), clofibrate was used to make the moiety for substitution at N3. This was done with a view to examine the effect of the* gem*-dimethyl on the overall activity of the resulting molecule.

All the 20 synthesised thiazolidin-4-ones were evaluated for glucose uptake in an* in vitro* system using the isolated rat diaphragm. In this experiment, compounds** 2e** and** 3a** significantly raised the amount of glucose uptake by the tissue both in the absence and presence of external insulin. This indicated the potential of the theses compounds in sensitizing the tissues for the external insulin. Hence these two compounds were chosen to study their effect on a diet-induced model of insulin resistance. Both compounds have the same p-methoxyphenyl moiety attached to the C2 of the thiazolidine ring. Compound** 3a** has a* gem*-dimethyl group instead of methylene in the substituent at N3.

Apart from multiple risk factors, diet-induced metabolic abnormalities contribute to the development of insulin resistance and *β*-cell failure in type-2 diabetes [16]. Early detection and appropriate treatment are considered beneficial for correcting the abnormality. Among the various animal models, induction of diabetes through diet provides more resemblance to human type-2 diabetes. Chronic intake of diet with high sucrose content has been reported to favour the development of insulin resistance [16]. Similarly in our study, mice fed a high-sucrose diet for six months developed metabolic abnormalities like hyperglycemia, hyperinsulinemia, hypertriglyceridemia, hypercholesterolemia, and hypoadiponectinemia. Hyperglycemia and hyperinsulinemia suggest the inability of insulin to sensitize the tissue for glucose uptake, allowing glucose to be diverted toward lipogenesis. This led to hyperlipidemia and finally to insulin resistance. In addition, adiponectin and leptin, the adipokines secreted from white adipose tissue (WAT), are reported to be involved in the metabolism of glucose and lipid [27]. In our study, we observed that HCD caused a reduction in plasma adiponectin level and an increase in the size of adipocytes. However, it did not cause any change in plasma leptin levels. Thus, we found a correlation between the adiponectin level and size of adipocytes in HCD model. Further, OGTT results from HCD-fed mice correlated well with glucose intolerance, hyperinsulinemia, and hypoadiponectinemia. The authenticity of the model was validated by the effect of pioglitazone, which was able to correct the metabolic abnormalities. Neither test compounds (**2e** or** 3a**) attenuated hypoadiponectinemia. However, they corrected the impaired glucose tolerance and insulin resistance in mice. Compounds** 2e** and** 3a**, by their ability to enhance glucose uptake and to sensitize the tissue for available insulin, reduced hyperinsulinemia and raised the leptin levels. This would have resulted in better glucose utilization by peripheral tissue. Thus, these compounds reduced the metabolic abnormalities like hyperglycemia, hypertriglyceridemia, and hypercholesterolemia. Prospective studies involving direct assessment of insulin sensitivity in OGTT are required to analyze glucose stimulated insulin secretion (GSIS) in presence of compounds to establish the mechanistic role of the test compounds on insulin signalling.

Histological investigation showed no change in liver architecture in the various treatment groups. In HCD control mice, there was an increase in the size of adipocytes in white adipose tissue (WAT), which was reversed by compound** 3a** and pioglitazone treatment. However, only pioglitazone-treated animals showed positive correlation between reduced size of the cells and correction of hypoadiponectinemia. Apart from correction of hypoadiponectinemia, pioglitazone raised the peripheral leptin levels, which was also true in case of compounds** 2e** and** 3a** treatment. Among the tested compounds,** 3a** raised endogenous leptin levels four times more than HCD group. This finding points to the link between raised leptin levels and reduced adipocyte size caused by compound** 3a**. The pancreatic islets showed hyperplasia in pioglitazone and other treatment groups. Pioglitazone is a PPAR-*γ* agonist. This could have been responsible for the proliferation of beta cells of pancreas. The test compounds are thiazolidin-4-ones with some similarity to thiazolidinediones. It is possible that hyperplasia observed in compound** 3a**-treated animals might have been due to some agonistic activity on PPAR-*γ* receptors. This needs to be investigated through relevant assay.

Oxidative stress has been implicated in the occurrence of diabetes and compounds reducing the oxidative stress have beneficial role in correcting glucose intolerance and insulin resistance in diabetes [18, 28]. The test compounds and pioglitazone reversed the depletion of endogenous antioxidant enzymes such as GSH, CAT, SOD, and GST. Further, they reduced malondialdehyde levels. This suggests the inhibitory effect of these compounds on oxidative stress.

Leptin serves as an insulin-sensitizing factor in the whole body [29]. However, hyperleptinemia in the obese mouse and human is a sign of leptin resistance, where increased leptin levels are caused by disturbed homeostasis, arising from leptin receptor mutation, ageing or obesity [29]. Thus, hyperleptinemia further worsens impaired insulin action in pathological condition. In these conditions, exogenously administered leptin does not improve glucose tolerance and insulin sensitivity. In our study, diseased animal (HCD fed mouse)* per se* did not develop hyperleptinemia, which might be the sign of early stage of metabolic abnormality associated with insulin resistance. At this state, test compounds as well as pioglitazone reversed the elevated glucose, TG, TC, and insulin levels while facilitating leptin profile along with insulin sensitivity. Subsequently, compound** 3a** treatment resulted in increased energy expenditure demonstrated by elevated food efficiency ratio (FER). However, reduction in body weight among the treated groups was not found proportionate to the circulating leptin levels, which suggests that increased leptin level in peripheral blood is not sufficient to induce a proportionate reduction on body weight. A few questions remain unanswered such as (i) whether the compounds directly raised the leptin levels or are the results due to indirect impact on metabolic signaling? (ii) In metabolic disorder combined with hyperleptinemia, how do these compounds affect leptin signaling? Future studies are needed to address these issues.

No single mechanism would suffice to explain the beneficial effects of the test compounds. They do not seem to act through insulinotropic activity unlike the sulfonylureas. They have no significant effect on adiponectin levels, ruling out any involvement of this mechanism. The increase in the level of serum leptin might point to the involvement of leptin in the antihyperlipidemic and antidiabetic potentials of these molecules.

In conclusion, thiazolidin-4-one derivatives act through multiple mechanisms to correct the metabolic abnormalities in type-2 diabetes. In the present work, compounds** 2e **and** 3a** were found to be the most effective test compounds to ameliorate insulin resistance and development of type-2 diabetes.

## Figures and Tables

**Table 1:** List of the twenty synthesized thiazolidin-4-ones.

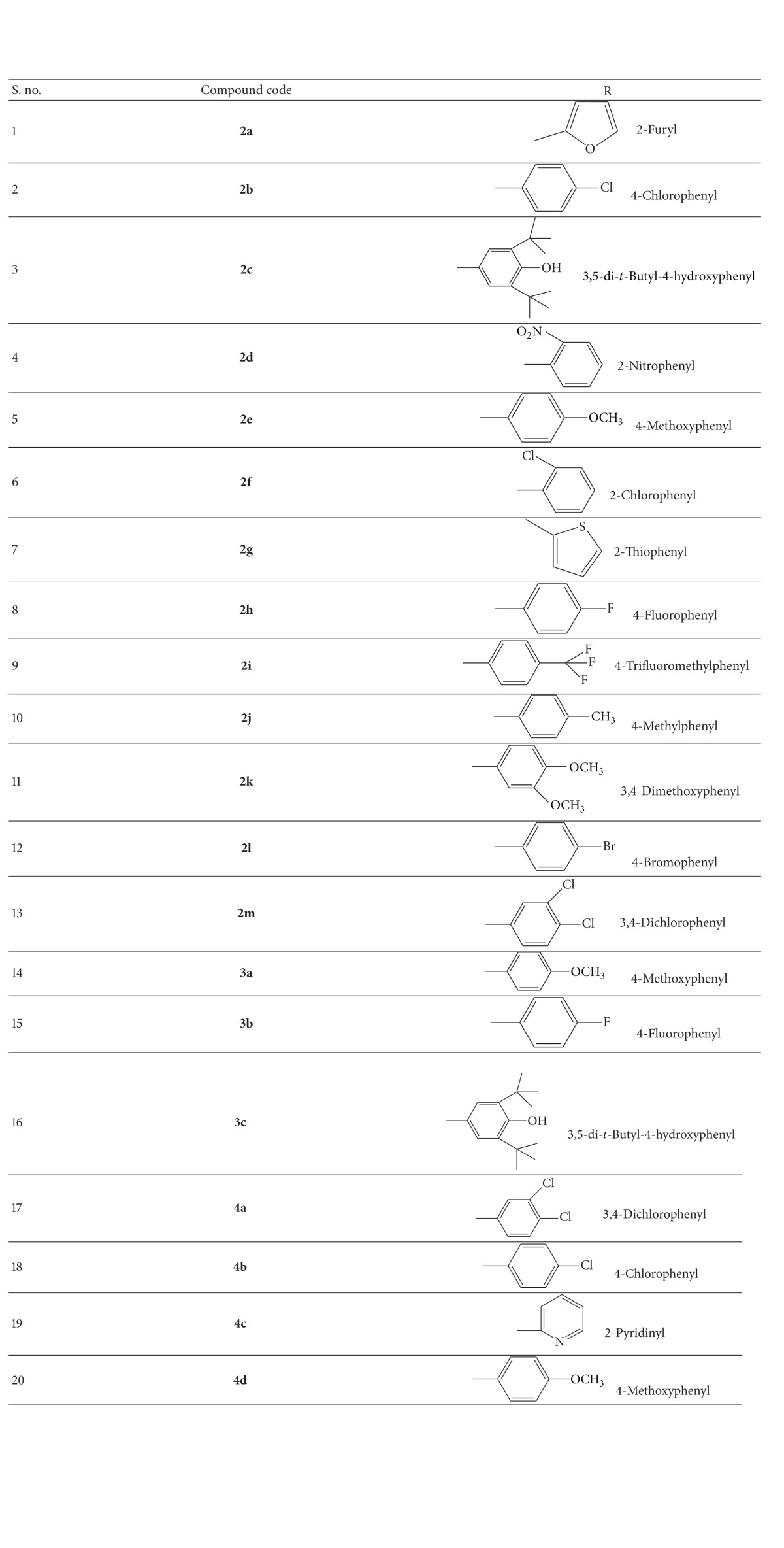

**Table 2:** Composition of high carbohydrate diet (HCD).

Sl. no.	Contents	Percent (w/w)
1	Cholesterol	2
2	Sucrose	55
3	Lard	3
4	Cellulose	3.50
5	L-cysteine	0.25
6	Choline bitartrate	0.50
7	DL-methionine	0.25
8	Vitamin and mineral mixture	0.10
9	Normal pellet diet	35.40

**Table 3:** Effect of thiazolidin-4-ones on *in vitro* glucose uptake in absence and presence of insulin.

S. no.	Groups	Glucose uptake (mg/g/45 min)	
		In absence of insulin	In presence of insulin
		Mean ± S.E.M. (*n* = 3)	Mean ± S.E.M. (*n* = 3)
1	Glucose control	8.09 ± 0.51	12.11 ± 1.34
2	Compound **2a**	1.89 ± 1.45	10.33 ± 1.21
3	Compound **2b**	6.19 ± 0.31	7.36 ± 0.25
4	Compound **2c**	10.12 ± 0.52	14.88 ± 0.86
5	Compound **2d**	9.84 ± 0.61	8.16 ± 0.11
6	Compound **2e**	19.00 ± 1.27^a^	23.42 ± 0.32^b^
7	Compound **2f**	6.03 ± 0.33	8.33 ± 0.15
8	Compound **2g**	6.45 ± 0.13	7.08 ± 0.19
9	Compound **2h**	7.61 ± 0.18	7.28 ± 0.38
10	Compound **2i**	5.55 ± 0.14	6.44 ± 1.19
11	Compound **2j**	9.54 ± 0.21	3.68 ± 1.79
12	Compound **2k**	5.82 ± 0.18	7.21 ± 0.15
13	Compound **2l**	6.43 ± 0.10	9.99 ± 0.10
14	Compound **2m**	8.12 ± 0.82	12.87 ± 0.15
15	Compound **3a**	20.44 ± 0.17^a^	25.01 ± 0.29^b^
16	Compound **3b**	7.77 ± 0.29	11.51 ± 0.02
17	Compound **3c**	12.24 ± 0.10	10.50 ± 0.81
18	Compound **4a**	7.53 ± 1.10	7.98 ± 0.83
19	Compound **4b**	6.10 ± 0.83	7.72 ± 0.21
20	Compound **4c**	0.97 ± 0.59	4.82 ± 0.55
21	Compound **4d**	3.77 ± 0.47	5.54 ± 0.49
22	Pioglitazone	13.37 ± 0.26^a^	25.23 ± 0.34^b^

a and b represent *P* < 0.05 as compared to glucose control in absence/presence of insulin, respectively.

**Table 4:** Effect of thiazolidin-4-ones on body weight and food efficiency ratio in mice.

	NPD	HCD	Pioglitazone	Compound **2e**	Compound **3a**
Final body weight (g)	38.0 ± 2.1	41.2 ± 2.2	38.2 ± 1.4	37.6 ± 1.2	38.2 ± 2.1
Food efficiency ratio	0.0025 ± 0.005	0.0083 ± 0.006	0.0020 ± 0.005	0.0043 ± 0.011	0.071 ± 0.010^a,b^

^a^Represents *P* < 0.05 as compared to NPD group (normal pellet diet).

^b^Represents *P* < 0.05 as compared to HCD group (high carbohydrate diet).

**Table 5:** Effect of thiazolidin-4-ones on liver antioxidant enzyme and malondialdehyde level.

Liver biomarkers	NPD	HCD	Pioglitazone	Compound **2e**	Compound **3a**
GSH (nmole/mg of protein)	55.8 ± 2.1	46.4 ± 2.1^a^	63.2 ± 1.5^b^	68.3 ± 2.0^b^	67.8 ± 4.3^b^
CAT (U/mg of protein)	89.2 ± 5.7	55.4 ± 3.1^a^	93.8 ± 1.0^b^	81.9 ± 2.4^b^	87.5 ± 7.4^b^
SOD (U/mg of protein)	186.8 ± 23.2	108.1 ± 11.2^a^	174.8 ± 14.4^b^	202.2 ± 4.7^b^	208.0 ± 15.4^b^
GST (U/mg of protein)	0.43 ± 0.05	0.37 ± 0.01^a^	0.73 ± 0.02^b^	0.70 ± 0.01^b^	0.72 ± 0.04^b^
MDA (nmole/mg of protein)	0.57 ± 0.06	1.0 ± 0.33^a^	0.46 ± 0.01^b^	0.40 ± 0.01^b^	0.41 ± 0.02^b^

^a^Represents *P* < 0.05 as compared to NPD group (normal pellet diet).

^b^Represents *P* < 0.05 as compared to HCD group (high carbohydrate diet).
